# Kinematic parameters and metabolic power in elite soccer players: A small sided a large sided games comparison

**DOI:** 10.3389/fphys.2023.1150713

**Published:** 2023-04-07

**Authors:** Nemanja Zlojutro, Serdar Eler, Marko Joksimovic, Nebahat Eler, Saša Marković, Aleksandar Kukrić, Kosta Goranovic

**Affiliations:** ^1^ Faculty of Physical Education and Sport, University of Banja Luka, Banja Luka, Bosnia and Herzegovina; ^2^ Faculty of Sport Science, Gazi University, Ankara, Ankara, Türkiye; ^3^ Faculty of Sports and Physical Education, University of Montenegro, Niksic, Montenegro; ^4^ Vocational School of Physical Education and Sports, Bülent Ecevit University, Zonguldak, Zonguldak, Türkiye

**Keywords:** soccer (football), global positing system (GPS), acceleration, small sided games, duration

## Abstract

**Introduction:** The goal of this paper is to determine what happens in one minute (on average) in kinematic parameters and metabolic power in small sided games (SSG) (3v3; 5v5) and large sided games (LSG) (10v10) and in which games kinematic parameters and metabolic power are best developed.

**Methods:** The participants of this study were 22 professional football players, height 182.95±6.52 cm, mass 77.17±8.21 kg, body mass index (BMI) 22.97±1.47 kg/m2, body fat 9.85±2.55 %, aged 27.1±5.4 yrs, who played in the Premier League of Bosnia and Herzegovina. Data total distance (TD), maximum speed (MS), number of accelerations (nAcc), number of decelerations (nDec), number of sprints (nS), high intensity distance (Z4≥19.8 km/h), sprint distance (Z5≥25.2 km/h) and movements requiring a certain metabolic power (Pmet), were collected using a 20 Hz Global positioning system (GPS) system Pro2 (GPEXE, Exelio srl, Udine, Italy), on a total of 307 individual observations.

**Results:** The results showed that the average total distance was significantly higher in the 5v5 (135.16±18.78 m) and 10v10 (133.43±20.06 m) games (F=64.26, p<0.001) compared to the 3v3 (108.24±11.26 m). Furthermore, the values of the variables Z4 (8.32±3.38 m, F=97.59), Z5 (1.84±1.53 m, F=123.64), nS (0.13±0.10 n, F=96.14) as well as Maxspeed (27.06±1.90 km/h, F=139.33), are statistically significantly higher (p<0.001) in the 10v10 game compared to the other two game formats. The average number of nAcc (0.40±0.32 n, F=9.86, p<0.001) and nDec (0.62±0.36 n, F=6.42, p<0.001) is statistically significantly higher in the 5v5 game. The results showed that the 5v5 game is significantly more metabolically demanding Pmet (2.76±0.67 W•kg^−1^, F=66.08, p<0.001) compared to the other two game formats.

**Discussion:** The data presented in this paper can be used as a basis for the construction of specific exercises based on kinematic and physiological requirements, and for planning and programming microcycles in football.

## Introduction

Sports with a ball, such as football, are complex adaptive systems that enable the emergence of rich patterns of player movement in a constantly dynamically changing environment (Silva et al., 2016). In elite football, coaches are constantly looking for drills and modified games, which can contribute to improved physical, technical and tactical performances of the football players. Although the football match is played with 11 players per team, the SSG often includes <4 players per team (with or without a goalkeeper) on reduced pitch areas (Dellal et al., 2011). The SSG is a training method for which coaches consider to optimize training time and allows trainers to repeat the requirements as in the football match (William et al., 2018). This is the reason football coaches constantly used SSG to improve and maintain physical fitness including technical and tactical performance in elite football. Internal and external loading in SSG characterize to collect values of heart rate, movement demands, blood lactate, and rate of perceived exertion (Hill-Haas et al., 2011). Research has confirmed that the size of the playing area, the rules of the game and number of players all influence the acute physiological response (Hill-Haas et al., 2011; Casamichana et al., 2012). Also, the validity and reliability of kinematic parameters has been the subject of extensive research in recent years. In the scientific literature, you can find a large number of scientific research that prove the validity and reliability of kinematic parameters (Scott et al., 2016; Bastida-Castillo et al., 2018). It is not well understood what impact SSGs may have in the hours and days that follow. A greater understanding of this would be of interest to those responsible for the design of football training programs, given the possible influence that this may have on additional training sessions performed within the week. The metabolic power (Pmet) presented as a tool to estimate the energetic demands of variable-speed and accelerated/decelerated locomotion activities typically seen in football games. While it is difficult to measure directly the exact energy cost of changing speed, a metabolic power calculation based on a theoretical model has been used to estimate the energy cost of locomotion in football games (Polglaze and Hoppe, 2019). However, this model was questioned since it may underestimate the actual net energy demand of football - specific exercises. Additionally, the traditional speed-threshold approach was shown to provide a similar external load compared to Pmet (Dubois et al., 2017). Nevertheless, the metabolic power approach could capture the high—demanding locomotor activities independently of the actual speed registered by GPS, and it was shown to be a useful tool for the classification of the locomotion intensity in team sports (Riboli et al., 2020). The study carried out by (Manzi et al., 2014) presented evidence of the validity of this approach by stating a positive correlation between Pmet and aerobic fitness during elite football matches. Moreover, Pmet can be sensitive to decrements in running performance during competition (Malone et al., 2016) and it could be used to account for positional differences. A combination of the Pmet approach and traditional speed—threshold measurement is used to understand assessment of the intermittent running demands typically occurring in football games (Riboli et al., 2020), because, may help to plan the training sessions to condition the locomotor activities typically required during the official match and to optimize performance goals (Martin-Garcia et al., 2018). The goal of this paper is to determine what happens in 1 min (on average) in kinematic parameters and metabolic power in SSG (3v3; 5v5; 10v10) and in which games kinematic parameters and metabolic power are best developed.

## Materials and methods

### Participants and drills observations

The research included a sample of 22 professional football players FK Borac, Banja Luka, height 182.95 ± 6.52 cm, body mass 77.17 ± 8.21 kg, BMI 22.97 ± 1.47 kg/m2, body fat 9.85% ± 2.55%, age 27.1 ± 5.4 years. A total of 307 individual drill observations were undertaken on outfield players (goalkeepers were excluded). The football players participated in this research are competing in the Premier league, highest ranked competition of Bosnia and Herzegovina. The testing was done in 2020/2021. To be qualified to participate in the research, the players should satisfy the following criteria: that the players have been on the first team for at least 6 months, that all players have gone through a preparatory period with the team, without injuries in the last 6 months, that they have played one half-season before testing. The footballers were excluded from the testing in case: football players in the recovery phase from some form of acute or chronic injuries, football players who did not complete the entire preparatory period. All the players were informed about the purpose and the goal of this research and the procedure was explained to them. The club president, main coach and all the players signed the paper accepting participation in the test. The research was approved by the Ethics Commission of the Faculty of Sports and Physical Education, University of Banja Luka in accordance with the Declaration of Helsinki. The players were instructed not to consume performance enhancing substances such as creatine, ribose, *etc.* (coffee was limited to 1 cup) prior to tests, not to engage in high intensity physical activity 24 h prior to the tests (Tatlicioglu et al., 2019).

### Data collection

The players’ physical activity during each training sessions were monitored using portable global 20 Hz GPS system Pro2 (GPEXE, Exelio srl, udinese, Italy). This version of the SPI Pro (6 g tri-axial accelerometer sampling at 20 Hz integrated; size = 48 × 20 × 87 mm; mass = 76 g) provides raw position, velocity and distance data at 20 Hz (20 samples per second). For the purpose of this study, every three raw data points were averaged to provide a sampling frequency of 5 Hz (Gaudino et al., 2013). A particular vest was tightly fitted to each player, placing the receiver between the scapulae. All devices were always activated 15-min before the data collection to allow acquisition of satellite signals (Maddison and Ni Mhurchu, 2009). The minimum acceptable number of available satellite signals was 8 (range 8–11) (Varley et al., 2012). Data was eliminated on days when the satellite signal was below this value. In addition, in order to avoid inter-unit error players wore the same GPS device for each training sessions (Buchheit et al., 2014). This type of system has previously been shown to provide valid and reliable estimates of instantaneous velocity during acceleration, deceleration, and constant velocity movements (Varley et al., 2012). This instrument has previously been used in order to quantify the number of accelerations during elite Australian football matches (Varley and Aughey, 2013). However, as 5 Hz GPS may slightly underestimates instantaneous velocity during acceleration or high speed movements any reported values in this investigation are the minimum of what a player would actually undertake during the analysed drill (Varley et al., 2012). Through the use of this instrument drill duration, total distance covered and distance covered in the different speed categories was calculated using a custom Excel spreadsheet from instantaneous raw data of time, speed and distance available from the GPEXE cloud (GPEXE, Exelio srl, Udine, Italy). In the same program instantaneous acceleration values were calculated by dividing the change in speed by time. Finally, the mathematical model proposed by di Prampero et al. (2005) were also integrated in the custom spreadsheet in order to calculate total estimated energy expenditure, average metabolic power, and distance covered in different metabolic power categories as reported in previously studies using GPS technology.

### Study procedures

Kinematic parameters, total distance passed (TD), maximum speed (MS), acceleration numbers (nAcc), deceleration numbers (nDec), number of sprints (nS), number of jumps (nJumps), distance (Z4), sprint distance (Z5) and movements requiring a certain metabolic power (Pmet), were obtained using a 20 Hz GPS system Pro2 (GPEXE, Exelio srl, Udine, Italy). Studies, (Nagahara et al., 2017; Hoppe et al., 2018), confirmed the reliability and validity of the application of the GPS system in science and practice. The GPEXE unit determined acceleration as any change in speed of movement by 2.5 m/s for a duration of 0.5 s, while it determined deceleration as any change in speed of movement by −2.5 m/s for a duration of 0.5 s. Furthermore, (Z4) are registered as high-intensity running ≥19.8 km/h for a minimum duration of 0.5 s, (Z5) sprint distance ≥25.2 km/h for a minimum duration of 0.5 s and the number of sprints as all movements at a speed ≥25.2 km/h for a minimum duration of 1 s. Regarding predicted metabolic parameters, average metabolic power (Pmet) was calculated (di Prampero et al., 2005). Pmet categories were defined as: distance covered (m) at high power (HP; from 20 to 35 W kg^-1^), elevated power (EP; from 35 to 55 W kg^-1^) and maximal power (MP; >55 W kg^-1^) (di Prampero et al., 2005; Gaudino et al., 2013). Total distance covered at high Pmet (TP; >20 W kg^-1^) was also analyzed as an indicator of the high intensity distance covered (Gaudino et al., 2013; Gaudino et al., 2014a). Jump calculation was performed based on the movement of the gyroscope integrated inside the GPS unit. A jump is software-determined as any movement of the GPS unit in the vertical direction over 20 cm.

### Small-sided games (SSG) and large sided games (LSG)

In this research (SSG) were analyzed in three different formats (small 3v3+GK, medium 5v5+GK and large 10v10 + GK) for the development of functional capacities in specific conditions during the training process in the 2020/21 season. It should be emphasized that the training units in which auxiliary games were conducted represented the most intense loads during the microcycle and were conducted 72 h after the competitive match. The training units consisted of: standard warm-up for 20 min, elementary technique exercises for 10 min, possession of the ball for 10 min, and after that SSG. All training units were realized in the afternoon hours on a surface with natural grass in optimal weather conditions. SSG and LSG were determined by the number of players, the duration of the game, the number of sets, the length of the break between sets and the dimensions of the playing field (Verheijen, 2014) which is shown in Table 1. All SSG and LSG were realized by playing between two standard-sized goals with goalkeepers (GK) in goal. Modified football rules were applied: the player was limited to a maximum of 3 touches with the ball, otherwise the ball went to the opposing team while the goalkeeper had a maximum of 3 s to put the ball into play. The ball going out or a goal-out was determined by throwing the ball into play by the goalkeeper in relation to which team the ball went. It is very important to emphasize that the assistant coaches stood around the field for playing with balls, they controlled the speed of putting the ball into the game in case the ball goes out or a goal out and verbally encouraged the players to high intensity of the game itself. All players were familiar with the experimental and training procedures and during training they always wore the same GPS unit to reduce measurement error. Also, only the data of players who were completely physically healthy and who performed all the scheduled series in a specific game format for the given training were included in the analysis.

**TABLE 1:** Small sided games and large sided games.

Game format	Game duration (min)	Pause between sets(min)	FieldDimensions (m)	Area of the playingField (m^2^)
**3v3+GK**	2 × 6 × 1 min	2	30 x 18	600
**5v5+GK**	4 x 5 min	2	50 x 30	1500
**10v10+GK**	3 x 12 min	2	100 x 60	6000

### Data analysis

The normality of the distributions was confirmed by the Kolmogorov-Smirnov test, and the data are presented as the means ± standard deviations. Differences among 3 different formations SSG by 1 min play were analyzed by one-way analysis of variance, while the consecutive LSD *post hoc* test was used to analyze the diffrences across variables. The statistical procedures were executed on SPSS software (version 26.0, IMB, United States) for *p* < 0.05.

## Results

Table 2 present the descriptive parameters of the kinematic analysis of 3 different support game formats (small 3v3 + GK, medium 5v5 + GK and large 10v10 + GK) based on 1 min of play (average).

**TABLE 2:** Descriptive statistics of variables based on 1 min of play in three different game formats.

Variables	SSG and LSG	Mean ± Std.Dev	95% CI
	3v3	108.24 ± 11.26	105.65–110.84
TD	5v5	135.16 ± 18.78	132.27–138.04
(m)	10v10	133.43 ± 20.06	128.53–138.32
	3v3	2.71 ± 1.59	2.34–3.08
Z4 (m)	5v5	3.26 ± 2.84	2.82–3.69
	10v10	8.32 ± 3.38	7.49–9.14
	3v3	0.98 ± 0.26	0.03–0.16
Z5 (m)	5v5	0.109 ± 0.46	0.03–0.18
	10v10	1.84 ± 1.53	1.47–2.22
	3v3	0.38 ± 0.18	0.34–0.43
AccEvents	5v5	0.40 ± 0.32	0.35–0.46
(n)	10v10	0.23 ± 0.12	0.21–0.27
	3v3	0.55 ± 0.22	0.51–0.61
DecEvents	5v5	0.62 ± 0.36	0.56–0.68
(n)	10v10	0.46 ± 0.17	0.42–0.51
	3v3	1.88 ± 0.37	1.80–1.97
Metpower	5v5	2.76 ± 0.67	2.65–2.86
(W∙kg^-1^)	10v10	2.07 ± 0.62	1.92–2.23
	3v3	23.60 ± 1.84	23.17–24.03
Maxspeed	5v5	22.05 ± 2.22	21.71–22.39
(km/h)	10v10	27.06 ± 1.90	26.60–27.53
	3v3	0.53 ± 0.31	0.46–0.60
Jumps	5v5	0.34 ± 0.36	0.29–0.40
(n)	10v10	0.07 ± 0.09	0.05–0.10
	3v3	0.01 ± 0.04	0.00–0.02
Sprints	5v5	0.01 ± 0.46	0.00–0.02
(n)	10v10	0.13 ± 0.10	0.11–0.16

Legend: TD, total distance; Z4, distance 4 (≥19,8 km/h); Z5, distance 5 (≥25.2 km/h); AccEvents, acceleration; DecEvents, deceleration; Metpower, Metabolic power; MaxSpeed, maximal speed.

Anova shows a statistically significant difference (*p* < 0.05) in all compared variables for all three game formats (Table 3). The analysis showed that the average distance covered in 1 min is significantly higher in the medium 5v5 (135.16 ± 18.78 m) and large 10v10 (133.43 ± 20.06 m) game formats (F = 64.26, *p* < 0.001) compared to the small 3v3 (108.24 ± 11.26 m) game format. However, it is very interesting that the statistical analysis did not show a statistically significant difference between the medium and large format of the game when it comes to the average distance passed in 1 min. Furthermore, the results show that the values of the variable (Z4 ≥ 19.8 km/h) obtained in the 10v10 game (8.32 ± 3.38 m, F = 97.59, p=<0.001) are significantly different compared to the values obtained in the 3v3 format (2.71 ± 1.59 m) and 5v5 (3.26 ± 2.84 m), while no statistically significant difference was obtained between the 3v3 and 5v5 games. The greatest distance in the Z5 sprint (≥25.2 km/h) was also achieved by the players in the 10v10 game (1.84 ± 1.53 m, F = 123.64, *p* < 0.001), which is a significantly higher result compared to this kinematic parameter achieved in the 3v3 and 5v5 games, with the fact that no statistically significant difference was observed between the small and medium format of the game. The average number of accelerations made by the players during 1 min is statistically significantly higher in 3v3 (0.38 ± 0.18) and 5v5 (0.40 ± 0.32, F = 9.86, *p* < 0.001) than in 10v10 games (0.23 ± 0.12). The results further show that there was a significantly higher average number of decelerations in the medium 5v5 game format (0.62 ± 0.36 n, F = 6.42, *p* < 0.001) compared to the small and large game formats. The statistical parameters showed that there is no significant difference between the small (3v3) and medium (5v5) game formats. Energy requirements assessed based on the Metpower variable showed that the medium 5v5 game format was significantly more metabolically demanding (2.76 ± 0.67 W kg^-1^, F = 66.08, *p* < 0.001) compared to the other two game formats, while between the small 3v3 (1.88 ± 0.37 W kg^-1^) and large 10v10 (2.07 ± 0.62 W kg^-1^) game format had no statistically significant differences. The average maximum running speed was statistically significantly higher in the large game format 10v10 (27.06 ± 1.90 km/h, F = 139.33, *p* < 0.001) compared to the remaining two game formats, however, it is interesting to note that the players in the small game format 3v3 (23.60 ± 1.84 km/h) achieved a statistically significantly higher average maximum speed compared to the medium format of the 5v5 game (22.05 ± 2.22 km/h).

**TABLE 3:** Differences in kinematic parameters and metabolic power.

Variables	Mean ± Std.Dev			F	Sig
	3v3	5v5	10v10		
TD (m)	108.24 ± 11.26	135.16 ± 18.78†	133.43 ± 20.06†	64.268	.**001**
Z4 (m)	2.71 ± 1.59	3.26 ± 2.84	8.32 ± 3.38†‡	97.590	**.001**
Z5 (m)	0.98 ± 0.26	0.109 ± 0.46	1.84 ± 1.53†‡	123.64	**.001**
Accevents	0.38 ± 0.18†	0.40 ± 0.32‡	0.23 ± 0.12	9.868	**.001**
(n)					
Decevents	0.55 ± 0.22	0.62 ± 0.36†‡	0.46 ± 0.17	6.423	**.002**
(n)					
Metpower (W∙kg-1)	1.88 ± 0.37	2.76 ± 0.67†‡	2.07 ± 0.62†	66.088	**.001**
Maxspeed	23.60 ± 1.84†	22.05 ± 2.22	27.06 ± 1.90†‡	139.331	**.001**
(km/h)					
Jumps (n)	0.53 ± 0.31†‡	0.34 ± 0.36†	0.07 ± 0.09	37.54	**.001**
Sprints (n)	0.01 ± 0.04	0.01 ± 0.46	0.13 ± 0.10†‡	96.149	**.001**

Legend: TD, total distance; Z4, threshold of zone 4; Z5, threshold of zone 5; AccEvents, acceleration; DecEvents, deceleration; Metpower, metabolic power; MaxSpeed, maximal speed.

The average number of jumps was significantly higher in the small game format 3v3 (0.53 ± 0.31 n, F = 37.54, *p* < 0.001) during 1 min of the game, compared to the other two games, while between the medium 5v5 (0.34 ± 0.36 n) and the large 10v10 (0.07 ± 0.09 n) game format confirmed a statistically significant difference. However, players achieved a significantly higher average number of sprints in the large 10v10 game format (0.13 ± 0.10 n, F = 96.14, *p* < 0.001) compared to the small 3v3 and medium 5v5 game formats, while no statistically significant difference was found between them.

## Discussion

The goal of this paper is to determine what happens in 1 min (on average) in kinematic parameters and metabolic power in SSG (3v3; 5v5; 10v10) and in which games kinematic parameters and metabolic power are best developed. According to the author’s knowledge so far, this is the first study that analysed what happens during 1 min (on average) in kinematic parameters and metabolic power in SSG. Small sided games are used in football training both for the development of technical-tactical skills and for developing performance, and have become one of the most popular training methods at all ages and levels. Exercises of this type put players in situations that closely resemble those they will encounter in real game conditions, and reproduce many of the physical, physiological and technical demands of competitive football (Castellano and Casamichana, 2013). Different variants of SSG (3v3, 5v5, 10v10) have a different impact on the development of certain kinematic parameters and metabolic power. The field size is considered a key factor in football training because the players' density conditions internal and external load (Sannicandro, 2021). The results obtained in this study indicates that in the 5v5 game the highest total distance passed was recorded (135.16 ± 18.78 m) compared to 3v3 and 10v10. In contrast to the results in this study, Guard et al. (2021), in a 5v5 game recorded a total distance of (242 m). Dellal et al. (2011) in the 3v3 game recorded values of (315 m) in elite soccer players, while in amateurs they recorded a value of (272 m). The results of the total distance obtained in this paper are significantly lower compared to the results obtained by (Dellal et al., 2011; Sannicandro, 2021) The reason for this is that in our study in SSG, the goalkeeper was used in all games, and the results represented average values during 1 min. Research indicates that the results obtained in SSG with goalkeepers lead to a lower heart rate and a lower total distance traveled by the players. Also, in SSG with a goalkeeper, players try to organize their team’s defense to protect their goal, which affects the total distance during the game (Mallo and Navarro, 2008), which is confirmed by our results obtained in all SSGs. In practical terms, the results of this study indicate that coaches can adjust the intensity of training by varying the size of the field. In general, the dimensions of the field can change the kinematics of movement in relation to each player. Basically, the space for possession of the ball and the execution of actions are directly related to the space between the players, as well as the free space for decision-making. In this sense, the player can be limited by the dimensions of the field, causing changes in physiological and kinematic parameters. Smaller spaces can encourage more stops, changes in movement or acceleration. On the other hand, larger dimensions of the field can allow players more time to move, to perform their actions planned and with more space (Tessitore et al., 2006). The total high-intensity running distance and sprints is indicated as a key factor for success in football match performance in addition to the technical skills to maintain greater ball possession, the total distance covered with ball possession, and the tactical behaviors (Riboli et al., 2022). The results of this study indicate that in variable Z4 (≥19.8 km/h) and Z5 (≥25.2 km/h) in the 10v10 game recorded higher values compared to the medium and small format games. The reason for such results is that the players had modified rules, the goalkeeper could keep the ball in his possession for a maximum of 3 s, while the players had the right to three contacts with the ball. The results in our study are justified by the research carried out by Jake et al. (2012), stating that the modified rules of the game significantly affect the intensity of game in SSG. On the other hand; Dellal et al. (2011), in their research state that free play in SSG leads to a greater number of duels but a reduced number of sprints and high-intensity running compared to one and two-touch play. The reason for the reduced intensity of running in the game 3v3 and 5v5 is the presence of the goalkeeper and the size of the field on which SSG is implemented (Sassi et al., 2004; Mallo and Navarro, 2008; Koklu et al., 2011). In large sided games, di Prampero et al. (2005), noted an increase in physiological and kinematic parameters. This contradiction may be due to the inclusion of goalkeepers changing the physiological and tactical behavior of outfield players, as it is possible that some players were more motivated than others. Therefore, the goal of scoring goals and at the same time protecting one’s own goal imposed greater physiological and kinematic activities on the players (Spalding et al., 2004). The average number of accelerations is significantly higher in the 3v3 and 5v5 games compared to the 10v10 game, while the number of decelerations is the highest in the 5v5 game compared to the 3v3 and 10v10 games. The results of this study are similar to another study by SSG in football (Guard et al., 2022). As stated by Guard et al. (2022), the higher loading of kinematic parameters (accelerations and decelerations) in the team that is not in possession of the ball, may be the result of higher average speed, acceleration and deceleration compared to the team that is in possession of the ball, creating a greater degree of consumption of anaerobic and aerobic energy, trying to regain possession of the ball. On the other hand, the distance traveled and the frequency of efforts performed for acceleration and deceleration are not feasible on small game formats. The reason for the large number of accelerations and decelerations is mainly due to the limited space where players, especially in the central areas, try to move away from the opponent and find space to receive the ball. Our statements are confirmed by the research of Seifert et al. (2013), in which it is stated that players can trigger powerful accelerations in their games, but are quickly hindered by the boundaries of the field. On the other side, player movements can be the result of continuous functional adaptation arising from game design to maximize success. According to the results of previous studies (Dellal et al., 2011; Hill-Haas et al., 2011; Castellano et al., 2013), the average energy consumption (Pmet) was higher as the surface of the field increased, the number of players decreased, or the number of players who had limited. The results of our study are not in agreement with previous studies because the higher energy consumption was recorded in the medium format 5v5 game. This suggests that small game areas stress different physiological components of performance compared to large areas, which cannot be detected by measuring distance traveled and speed achieved (Gaudino et al., 2014b). When it comes to maximum speed, the results obtained in this research indicate that the highest speed was recorded in the 10v10 game. On the other hand, a higher maximum speed was achieved in the 3v3 game compared to the 5v5 game. In previous research, it has been found that SSG with goalkeepers provides an environment where the total distance increases with more players and available playing space, which is explained by the higher average speed of players trying to find space away from their opponents. The higher average maximum speed also makes it difficult to accelerate and decelerate from a higher speed, given that the movement speed is already relatively high and the limited space in the SSG does not allow for pronounced high-speed activity. Therefore, it can be suggested that SSG appear to be highly contextual in how their design affects individual physical and subjective outcomes (Guard et al., 2021). Research comparing SSG with and without a goalkeeper indicates that games without a goalkeeper are physiologically more demanding in terms of kinematic parameters (Castellano and Casamichana, 2013). Identifying the most important kinematic predictors of jump performance allows coaches to monitor and strive to develop jump-specific performance (Murtagh et al., 2017). The results obtained in this study indicate that the highest number of rebounds (on average) was obtained in small and medium format games (3v3 and 5v5) compared to the large sided game (10v10). The reason for the large number of jumps in the 3v3 and 5v5 games lies in the fact that the goalkeeper had to return the ball to the game with his hand, which led to a large number of jumps and aerial duels. Future research should be carried out with the restriction that the goalkeeper cannot give high balls, in order to get a precise insight into how many jumps are realized in SSG. On the other hand, elite football players who presented moderate improvements in vertical jump ability, performing jumps during the preparatory period in SSG on the small space, could directly transfer these results to the sprint results in SSG on the large format (Loturco et al., 2015) which justifies the results obtained in our study of the SSG sprint numbers on the large format game.

Given that this study did not analyze kinematic parameters by player positions, we can consider this as a lack of research. Future research should be conducted on a larger sample and perhaps players of different levels of competition. However, this study will also have a practical application in the programming of individual trainings based on the knowledge of the requirements of different SSG formats when it comes to kinematic and metabolic parameters and as a comparative basis for monitoring training of a similar orientation.

## Conclusion

These specific forms of the SSG format can be used for pre-season or in-season conditioning purposes for those players who are not exposed to regular match play and require a higher load and training volume during the training week. This is particularly relevant to the development of youngsters who are often asked to join first team training sessions and may not have had enough match exposure as seniors who are part of the starting team. The disadvantage of SSG is that not all players exercise at a similar intensity, with relatively large variations in physiological stress. In addition, according to the results of this study as well as previous literature, coaches should avoid involving goalkeepers during SSG and use only small goals to preserve player motivation and training intensity. Finally, based on the results, we can see that there is a significant difference in the manifestation of kinematic parameters, in relation to the game format. The results tell us that by adequate periodization of different SSG, we can induce different adaptive responses, and with the goal of better competitive performance, taking into account many factors that affect the performance of the games themselves. Given that the obtained results are shown per minute, we can have a more detailed insight into all phases of the game.

## Data Availability

The original contributions presented in the study are included in the article/supplementary material, further inquiries can be directed to the corresponding author.

## References

[B1] Bastida Castillo A. Gómez-Carmona C. D. De la cruz sánchez E. Pino-Ortega J. (2018). Accuracy, intra-and inter-unit reliability, and comparison between GPS and UWB-based position-tracking systems used for time–motion analyses in soccer. Eur. J. sport Sci. 18 (4), 450–457. 10.1080/17461391.2018.1427796 29385963

[B2] Buchheit M. Al Haddad H. Simpson B. M. Palazzi D. Bourdon P. C. Di Salvo V. (2014). Monitoring accelerations with GPS in football: Time to slow down? Int. J. Sports Physiology Perform. 9, 442–445. 10.1123/ijspp.2013-0187 23916989

[B3] Casamichana D. Castellano J. Castagna C. (2012). Comparing the physical demands of friendly matches and small-sided games in semi-professional soccer players. J. Strength Cond. Res. 26, 837–843. 10.1519/JSC.0b013e31822a61cf 22310516

[B4] Castellano J. Casamichana D. Dellal A. (2013). Influence of game format and number of players on heart rate responses and physical demands in small-sided soccer games. J. Strength Cond. Res. 27, 1295–1303. 10.1519/JSC.0b013e318267a5d1 22836601

[B5] Castellano J. Casamichana D. (2013). Differences in the number of accelerations between small-sided games and friendly matches in soccer. J. Sports Sci. Med. 12 (1), 209–210. 24137079 PMC3761762

[B6] Dellal A. Hill-Haas S. Lago-Penas C. Chamari K. (2011). Small sided games in soccer: Amateur vs. professional players’ physiological responses, physical, and technical activities. J. Strength Cond. Res. 25 (9), 2371–2381. 10.1519/JSC.0b013e3181fb4296 21869625

[B7] di Prampero P. E. Fusi S. Sepulcri L. Morin J. B. Belli A. Antonutto G. (2005). Sprint running: A new energetic approach. J. Exp. Biol. 208, 2809–2816. 10.1242/jeb.01700 16000549

[B8] Dubois R. Paillard T. Lyons M. McGrath D. Maurelli O. Prioux J. (2017). Running and metabolic demands of elite rugby union assessed using traditional, metabolic power, and heart rate monitoring methods. J. Sports Sci. Med. 16 (1), 84–92. 28344455 PMC5358036

[B9] Gaudino P. Alberti G. Iaia F. M. (2014a). Estimated metabolic and mechanical demands during different small-sided games in elite soccer players. Hum. Mov. Sci. 36, 123–133. 10.1016/j.humov.2014.05.006 24968370

[B10] Gaudino P. Iaia F. M. Alberti G. Hawkins R. D. Strudwick A. J. Gregson W. (2014b). Systematic bias between running speed and metabolic power data in elite soccer players: Influence of drill typefluence of drill type. Int. J. Sports Med. 35, 489–493. 10.1055/s-0033-1355418 24165959

[B11] Gaudino P. Iaia F. M. Alberti G. Strudwic A. J. Atkinson G. Gregson W. (2013). Monitoring training in elite soccer players: Systematic bias between running speed and metabolic power data. Int. J. Sports Med. 34, 963–968. 10.1055/s-0033-1337943 23549691

[B12] Guard A. McMillan K. MacFarlane N. (2021). Influence of game format and team strategy on physical and perceptual intensity in soccer small-sided games. Int. J. Sports Sci. Coach. 17 (5), 1109–1118. 10.1177/17479541211056399

[B13] Guard A. N. McMillan K. MacFarlane N. G. (2022). The influence of relative playing area and player numerical imbalance on physical and perceptual demands in soccer small-sided game formats. Sci. Med. Footb. 6 (2), 221–227. 10.1080/24733938.2021.1939408 35475753

[B14] Hill-Haas S. V. Dawson B. Impellizzeri F. M. Coutts A. J. (2011). Physiology of small-sided games training in football: A systematic review. Sports Med. 41, 199–220. 10.2165/11539740-000000000-00000 21395363

[B15] Hoppe M. Baumgart C. Polglaze T. Freiwald J. (2018). Validity and reliability of GPS and LPS for measuring distances covered and sprint mechanical properties in team sports. PLoS ONE 13 (2), e0192708. 10.1371/journal.pone.0192708 29420620 PMC5805339

[B16] Jake N. Tsui M. C. Smith A. W. Carling C. Chan G. S. Wong D. P. (2012). The effects of man-marking on work intensity in small-sided soccer games. J. Sports Sci. Med. 11, 109–114. 24149127 PMC3737838

[B17] Köklü Y. Asci A. Kocak F. U. Alemdaroglu U. Dundar U. (2011). Comparison of the physiological responses to different small-sided games in elite young soccer players. J. Strength Cond. Res. 25, 1522–1528. 10.1519/JSC.0b013e3181e06ee1 21399538

[B18] Loturco I. Pereira L. A. Kobal R. Zanetti V. Kitamura K. Abad C. C. (2015). Transference effect of vertical and horizontal plyometrics on sprint performance of high-level U-20 soccer players. J. Sports Sci. 33, 2182–2191. 10.1080/02640414.2015.1081394 26390150

[B19] Maddison R. Ni Mhurchu C. (2009). Global positioning system: A new opportunity in physical activity measurement. Int. J. Behav. Nutr. Phys. Activity 6, 73. 10.1186/1479-5868-6-73 PMC2777117 19887012

[B20] Mallo J. Navarro E. (2008). Physical load imposed on soccer players during small-sided training games. J. Sports Med. Phys. Fit. 48 (2), 166–171. 18427410

[B21] Malone S. Solan B. Collins K. Doran D. (2016). The metabolic power and energetic demands of elite Gaelic football match play. J. Sports Med. Phys. Fit. 57 (5), 543–549. 10.23736/S0022-4707.16.06233-2 27029959

[B22] Manzi V. Impellizzeri F. Castagna C. (2014). Aerobic fitness ecological validity in elite soccer players: A metabolic power approach. J. Strength Cond. Res. 28 (4), 914–919. 10.1519/JSC.0000000000000239 24345968

[B23] Martin-Garcia A. Gomez-Diaz A. Bradley P. S. Morera F. Casamichana D. (2018). Quantification of a professional football team's external load using a microcycle structure. J. Strength Cond. Res. 32 (12), 3511–3518. 10.1519/JSC.0000000000002816 30199452

[B24] Murtagh C. F. Vanrenterghem J. O’Boyle A. Morgans R. Drust B. Erskine R. M. (2017). Unilateral jumps in different directions: A novel assessment of soccer-associated power? J. Sci. Med. Sport 20 (11), 1018–1023. 10.1016/j.jsams.2017.03.016 28416354

[B25] Nagahara R. Botter A. Rejc E. Koido M. Shimizu T. Samozino P. (2017). Concurrent validity of GPS for deriving mechanical properties of sprint acceleration. Int. J. Sports Physiology Perform. 12 (1), 129–132. 10.1123/ijspp.2015-0566 27002693

[B26] Polglaze T. Hoppe M. W. (2019). Metabolic power: A step in the right direction for team sports. Int. J. Sports Physiol. Perform. 14, 1–5. 10.1123/ijspp.2018-0661 30732493

[B27] Riboli A. Coratella G. Rampichini S. Ce´ E. Esposito F. (2020). Area per player in small-sided games to replicate the external load and estimated physiological match demands in elite soccer players. PLoS ONE 15 (9), e0229194. 10.1371/journal.pone.0229194 32966305 PMC7510966

[B28] Riboli A. Olthof S. B. H. Esposito F. Coratella G. (2022). Training elite youth soccer players: Area per player in small-sided games to replicate the match demands. Biol. Sport. 39 (3), 579–598. 10.5114/biolsport.2022.106388 35959338 PMC9331353

[B29] Sannicandro I. (2021). Small-side games and size pitch in elite female soccer players: A short narrative review. J. Hum. Sport Exerc. 16 (2), S361–S369. 10.14198/jhse.2021.16.Proc2.20

[B30] Sassi R. Reilly T. Impellizzeri F. M. (2004). A comparison of small-sided games and interval training in elite professional soccer players. J. Sports Sci. 22, 562. 10.4324/9780203412992

[B31] Scott M. T. Scott T. J. Kelly V. G. (2016). The validity and reliability of global positioning systems in team sport: A brief review. J. Strength & Cond. Res. 30 (5), 1470–1490. 10.1519/JSC.0000000000001221 26439776

[B32] Seifert L. Button C. Davids K. (2013). Key properties of expert movement systems in sport: An ecological dynamics perspective. Sports Med. 43 (3), 167–178. 10.1007/s40279-012-0011-z 23329604

[B33] Silva P. Vilar L. Davids K. Araujo D. Garganta J. (2016). Sports teams as complex adaptive systems: Manipulating player numbers shapes behaviors during football small-sided games. Springer Plus 5, 191. 10.1186/s40064-016-1813-5 27026887 PMC4769238

[B34] Spalding T. W. Lyon L. A. Steel D. H. Hatfield B. D. (2004). Aerobic exercise training and cardiovascular reactivity to psychological stress in sedentary young normotensive men and women. Psychophysiology 41, 552–562. 10.1111/j.1469-8986.2004.00184.x 15189478

[B35] Tatlicioglu E. Atalag O. Kirmizigil B. Kurt C. Acar M. F. (2019). Side to side asymmetry in lower limb strength and hamstring-quadriceps strength ratio among collegiate American football players. J. Phys. Ther. Sci. 31, 884–888. 10.1589/jpts.31.884 31871371 PMC6879405

[B36] Tessitore A. Meeusen R. Piacentini M. F. Demarie S. Capranica L. (2006). Physiological and technical aspects of 6-a side soccer drills. J. Sports Med. Phys. Fit. 46 (1), 36–43. 16596097

[B37] Varley M. C. Aughey R. J. (2013). Acceleration profiles in elite Australian soccerfiles in elite Australian soccer. Int. J. Sports Med. 34, 34–39. 10.1055/s-0032-1316315 22895869

[B38] Varley M. C. Fairweather I. H. Aughey R. J. (2012). Validity and reliability of GPS for measuring instantaneous velocity during acceleration, deceleration, and constant motion. J. Sports Sci. 30, 121–127. 10.1080/02640414.2011.627941 22122431

[B39] Verheijen R. (2014). *The original guide to football periodisation: Always play with your strongest team* (Part 1). Amsterdam: World Football Academy BV.

[B40] William S. Turner A. N. Weston M. Russell M. Johnston M. J. Kilduff L. P. (2018). Neuromuscular, biochemical, endocrine, and mood responses to small-sided games’ training in professional soccer. J. Strength Cond. Res. 32 (9), 2569–2576. 10.1519/JSC.0000000000002424 29351166

